# It’s bulking season! Acute water ingestion increases lean soft tissue estimation via dual-energy X-ray absorptiometry

**DOI:** 10.1080/15502783.2025.2550196

**Published:** 2025-08-28

**Authors:** Jeffery L. Heileson, Tina E. Sergi, Delon D. Yadavongsy, Grant M. Tinsley

**Affiliations:** a Nutrition Services Department, Walter Reed National Military Medical Center, Bethesda, MD, USA; b Health, Human Performance, Recreation, Baylor University, Waco, TX, USA; c Department of Kinesiology and Sport Management, Texas Tech University, Lubbock, TX, USA

**Keywords:** Body mass, body composition, water intake, DXA

## Abstract

**Background:**

While fasting is commonly recommended prior to body composition assessment, the influence of recent water intake is less clearly established. The purpose of this study was to determine the relationship between acute water ingestion (AWI; dose-response [0 mL, 250 mL, 500 mL, 1000 mL] and relative amount [mL∙kg^−1^]) and lean soft tissue (LST) estimation via dual-energy x-ray absorptiometry (DXA) over time (0–90 min).

**Methods:**

Forty-six males and females completed body composition analysis via DXA at baseline (BL). Immediately following the DXA scan, participants were randomized to no water (control [CON], *n* = 11), 250 mL (*n* = 12), 500 mL (*n* = 11), or 1000 mL (*n* = 12) of water intake. Participants then had body composition analyzed immediately after consumption (IP), then at 30 min (T30), 60 min (T60), and 90 min (T90). Height (cm) was measured at BL and body mass (kg) was assessed at each timepoint. Correlations and linear regression were used to determine the relationship between relative amounts of water intake (mL∙kg^−1^) and LST. A mixed-model repeated measures ANOVA (Group: 4 levels, Time: 4 levels) was used for analysis of the change in LST. For significant differences (*p* < .05), a post hoc Bonferroni correction was applied to identify specific differences between time and groups.

**Results:**

Body mass increased across all water intake groups and was significantly higher than CON at all timepoints (*p* < .05). The change in LST was significantly different over time and between groups. Compared to BL, LST was significantly higher in the 500 (T90, *p* = .047) and 1000 mL (*p* < .001 at all timepoints) groups with no significant changes noted for CON or 250 mL. Between groups, the change in LST was greater at all timepoints in the 1000 mL group, except for T90. At T90, the change in LST was similar between 1000 mL and 500 mL (*p* = .26), 1000 mL was greater than CON (*p* < .0001) and 250 mL (*p* = .0003), whereas 500 mL was greater than CON (*p* = .009). Relative AWI significantly predicted the change in LST, regardless of timepoint analyzed (R^2^ = 0.167–0.342, *p* = .0002–.0117).

**Conclusions:**

AWI artificially increases LST estimates via DXA. Data suggests that the change in LST was significantly higher following a water bolus of 1000 mL through 90 min and 500 mL after 90 min. Relative to body mass, higher water intake, somewhat consistently, led to higher LST estimation, especially ≥9 mL∙kg^−1^. To protect against error, pre-assessment DXA guidelines should recommend water intake ≤250 mL in the 90-min prior to assessment.

